# An agency mapping of marginalized communities and aid providers during the COVID-19 pandemic in Malaysia

**DOI:** 10.12688/wellcomeopenres.17315.1

**Published:** 2021-11-25

**Authors:** Melati Nungsari, Chuah Hui Yin, Nicole Fong, Veena Pillai

**Affiliations:** 1 Massachusetts Institute of Technology, Cambridge, Massachusetts, USA; 2 Asia School of Business, Kuala Lumpur, Malaysia; 3 Diode Consultancy, Kuala Lumpur, Malaysia

**Keywords:** aid provision, agency, vulnerable groups, humanitarian work, public health, developing countries, emerging markets, social cognitive theory, triadic reciprocal causation model

## Abstract

**Background:** Given the urgent need for help amongst vulnerable populations throughout the coronavirus disease 2019 (COVID-19) pandemic, civil society organizations (CSOs) and members have stepped up to provide support for impacted communities. The process of responding to these urgent needs reflects the agency and resilience of civil society members in accessing or providing resources. There is still a lack of understanding of how is human agency exercised in the context of power imbalances. Such an understanding is important not only for creating an effective and inclusive aid delivery mechanisms but also improving preparedness for future public health and economic crises.

**Methods:** This study utilizes Albert Bandura’s social cognitive theory to comprehensively map the agency landscape of aid providers and marginalized populations during the first few months of the COVID-19 pandemic in Malaysia. Assuming that these populations’ main goals are access to aid while providers’ main goals are to provide aid, this study categorizes the different modes of agency involved and highlights environmental facilitators and constraints for each of these groups in achieving their goals. Data was collected through in-depth interviews with 34 participants. Using a hermeneutic content analysis based on a sample of 824 textual excerpts from the interviews, we explore the relationship between each component of the agency landscape to understand the relationships between them.

**Results:** We find that marginalized populations are often unable to achieve their goals despite clear intentions to survive. Additionally, we find that proxy agency is problematic for marginalized populations and characterize why this is the case.

**Conclusions:** Finally, we present policy recommendations which prioritise marginalized populations and their needs, while removing barriers to accessing aid.

## Introduction

The coronavirus disease 2019 (COVID-19) pandemic has caused unprecedented shocks globally. As of August 13 ^th^, 2021, over 205 million confirmed cases and 4.3 million deaths were reported ( WHO, 2021). Public health systems were overwhelmed by its rapid spread, with resources like ventilators and hospital beds stretched thin, forcing doctors to “play God” by deciding who got to live ( Dyer, 2021; Wikler, 2020). The socioeconomic impacts are equally devastating. Various forms of lockdown measures imposed to combat the pandemic have also led to negative repercussions for the global economy. Private sector businesses, particularly non-essential ones, were forced to shut either permanently or temporarily, which directly caused widespread unemployment or salary cuts ( Chetty *et al.*, 2020). This also exposed and exacerbated the vulnerability of marginalized populations, including low income communities, immigrants, racial and ethnic minorities who were already facing economic insecurity – they are disproportionately impacted by the health and socio-economic implications of the COVID-19 pandemic and containment measures that follow ( Clark *et al.,* 2020; Gaynor & Wilson, 2020; Kantamneni, 2020; World Bank, 2020).

In Malaysia, the first national lockdown, known as the Movement Control Order (MCO), was imposed on March 18 ^th^, 2020 ( Prime Minister’s Office of Malaysia, 2020). Despite more than a year in which multiple stages of the MCO were implemented, cases reached a record five-figure high in mid-July 2021 ( Dong *et al.*, 2020). The debate on lives versus livelihoods remains a conundrum for policymakers. The latest Household Income Estimates and Incidence of Poverty Report ( Department of Statistics Malaysia, 2021) reveals that Malaysian households experienced a 10% decline in average income in 2020. Both absolute and hardcore poverty rates increased from 2019 (from 5.6%–8.4% and from 0.4%–1%, respectively).

In order to cushion these socioeconomic impacts, the government launched eight major relief and stimulus programs amounting to RM530 billion (approximately USD125 billion) as of June 2021 ( Prime Minister’s Office of Malaysia, 2021). While these initiatives are commended, some of the most vulnerable populations have fallen through the cracks. The initiatives are seen to be discriminatory in nature and do not cover migrant workers ( Verghis *et al.*, 2021). Cash transfers, for instance, are only a band-aid solution that may leave out people from rural communities who lack bank accounts or digital literacy, as well as informal workers who have no proof of income ( Flanders *et al.,* 2020; Lim, 2020).

Given the urgent need for help among vulnerable populations, there has been an emergence of initiatives led by civil society organizations (CSOs) and members which aim to bridge the gap by providing support for impacted communities. These include distributing food aid packages, fundraising for medical equipment and mask-sewing projects, just to mention a few ( Abd Samat *et al.*, 2021; Achremowicz & Kamińska-Sztark, 2020; Serina, 2020). The process of responding to these urgent needs also reflects the agency and resilience of civil society ( Fransen *et al.*, 2021). Yet there is still a lack of understanding of this process – how is human agency exercised, especially in the context of power imbalances i.e., the dependency of marginalized populations on these aid providers? Such an understanding is important not only for creating an effective and inclusive aid delivery mechanism but also improving preparedness for future crises.

This study utilizes Bandura’s social cognitive theory and his model of triadic reciprocal causation (henceforth, Bandura’s triadic model) ( Bandura, 1986; Bergman *et al.*, 2019) to understand the agency of organizations or individuals involved in providing and receiving aid during the COVID-19 pandemic. The next section presents the theoretical framework of Bandura’s triadic model, which illustrates the complex interdependence between civil society, marginalized communities, enforcement authorities, policymakers and the environment in which the aid delivery mechanism is embedded, followed by our methodology and findings.

## Theoretical background

According to social cognitive theory ( Bandura, 1986; Bandura, 2001; Bandura, 2006), people “are agents of experiences rather than simply undergoers of experiences”. This marks a departure from the dualistic and linear model, which suggests that humans are simply the products of their environment. Instead, Bandura’s theory and model suggest that one’s actions are shaped by the interactions of intrapersonal, behavioural and environmental determinants. Here, individuals are able to influence their actions and choices by exercising personal agency, which exist in three modes (i.e., individual, proxy and collective). Exercising proxy agency means that one has to rely on others with the means or resources to achieve desired outcomes on their behalf, while collective agency involves collective and interdependent group efforts to reach targeted goals.

The exercise of personal agency is interdependent with environmental influences. Social cognitive theory distinguishes three environments (i.e., selected, constructed and imposed), which affect the exercise of personal agency while being shaped by human agency ( Bandura, 1997). Selected environments provide the most room to exercise personal agency, while imposed environments are the most restrictive, in which one’s full ability to exercise personal agency is limited. In some cases, people also exercise agency in environments which do not pre-exist, but rather, are built through concerted efforts ( *Ibid.*).

**Figure 1.**
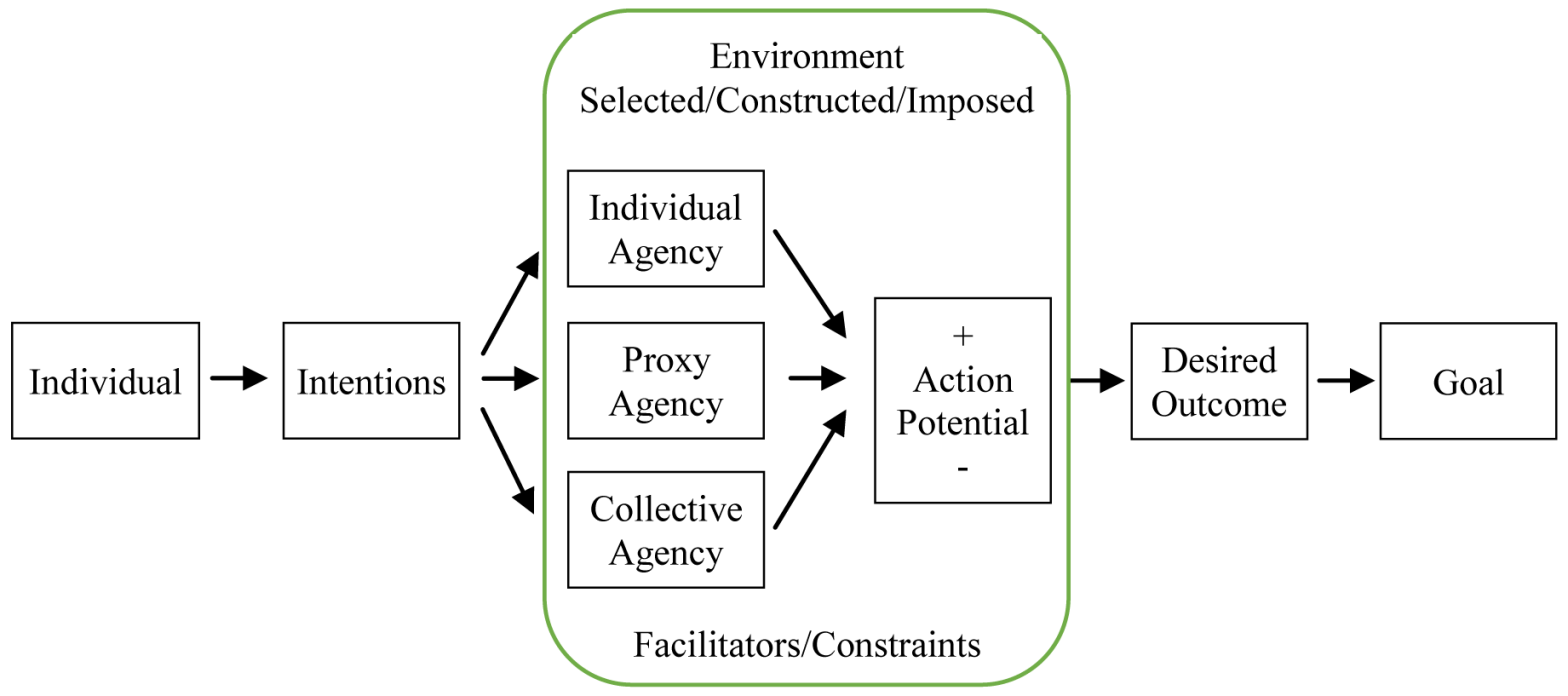
Personal agency, according to Bandura’s model of triadic reciprocal causation ( Bergman *et al.,* 2019).

Figure 1 below models individual intentions to achieve desired outcomes ( Bergman *et al.,* 2019). Through complex processes of intrapersonal deliberation, individuals assess how various environments facilitate or constrain their potential to act (i.e., positive and negative action potential), as well as how different modes of agency enable them to achieve their goals. Based on deliberations within environments, individuals can choose the mode of agency likeliest to secure a desired outcome in a specific context. An appropriate course of action is then selected and implemented.

In this context, the pandemic has caused unprecedented disruptions in almost all aspects of life, including work, education and mobility. The emergence of grassroots initiatives is an exercise of personal agency (i.e., people respond and adapt by mobilising available resources), which in turn translates to extensive aid distribution networks which provide emergency relief to marginalized communities. We decompose the agentive process by which aid is provided and received during the pandemic to understand: (1) the type of personal agency applied; (2) the environment in which they exercise their agency; (3) environmental constraints or facilitators of agency; as well as (4) positive and negative factors that could potentially enable or discourage them from exercising their agency.

In our context, we examine the agency landscape of marginalized populations. We split the dataset into data points relevant to aid providers or organizations, identified the modes of agency during this crisis as well as the environmental constraints and facilitators that either helped or impeded aid organizations and marginalized populations. Through a hermeneutic content analysis (HCA), we also examine how different parts of the Bandura agency landscape interact with each other and explore the reasons why relationships are the way they are.

## Methods

### Consent and ethics approval

The project was approved by two ethics review committees - the Ethics Consultative Services for Marginalised Population (ECS-MP) at the University of Malaya (UM) in Kuala Lumpur and the WHO’s Research Ethics Review Committee (WHO ERC). The dataset collected through this project was also used for another paper that examines ethics during the provision of aid ( Nungsari *et al.,* 2021a). Ethics approval was obtained on February 15 ^th^ and January 27 ^th^, 2021, respectively. ^2^ All interviews were conducted only after informed consent was given by the participants. Before the interviews, they were also informed of their agency in ending the interview at any time.

### Participant sampling and selection

Inclusion criteria were being above the age of 18 years, had the capacity to provide consent and belonged to one of the following categories:

- populations on the move (i.e., stateless persons, regular migrants, irregular migrants, refugees, asylum-seekers or undocumented persons);
- indigenous populations (i.e., Orang Asli populations in West Malaysia or Orang Asal populations in East Malaysia);
- CSO members or leaders who worked with these populations; or
- employers of members of these populations.

We recruited the participants through convenience sampling by disseminating information about the study and calls for participants through CSOs, their related networks and researchers’ professional and institutional networks, with an emphasis on the study’s voluntary nature. The first round of participants was gathered through email, social media and messaging apps such as WhatsApp. The remaining sample were recruited through snowball approach with their consent and they were contacted via the same methods after the existing participants shared their contacts with us.

The target sample size was originally 40–45 participants, i.e. roughly five to seven participants recruited from two categories: populations of the move and indigenous populations. The *a priori* sample size is set based on the complexity of the research questions and stratification factors of the sample. However, our reach and access to these communities was affected by the mobility restriction caused by strict lockdowns and digital divide in Malaysia. We approached 75 individuals in total, and ended up with 34 participants. ^3^ Another possible reason for low response rate is that since the data was collected during the lockdown, many organisations and individuals were still fully occupied with their work on the ground such as food aid provision. Among the participants, 31 participants are representatives of non-governmental organizations (NGOs) and businesses, while three of them are independent individuals from the two targeted categories. Table 1 provides more complete details on the participants. For example, Participant 1 is from a NGO in Sabah that provides aid to stateless persons, sex workers and sexual minorities. We stopped the interview as data saturation was achieved: namely, when the team started obtaining similar themes or findings from different participants.

**Table 1.** Details of the interviewees.

Participant Number	State	Community Served or a Part Of	Type of Organization
1	Sabah	Stateless, Sex Workers, Lesbian, Gay, Bisexual, Transgender, Queer and Others (LGBTQ+)	NGO providing aid
2	Klang Valley	Stateless, Indigenous, Unaccompanied Minor Refugees, Trafficked Victims	NGO providing aid, case management, education, and community placement
3	Klang Valley	Refugees	Individual
4	Sarawak	Indigenous, Women	NGO providing aid, case management
5	Klang Valley	Migrant, Refugees, Stateless	NGO providing aid, outreach programs, livelihood programs
6	Klang Valley	Refugees, Migrants	NGO providing aid, case management
7	Klang Valley	Refugees	Individual
8	Klang Valley	Refugees	NGO providing aid, case management, outreach programs, livelihood programs
9	Sarawak	Indigenous, Urban Poor	NGO providing aid, case management
10	Klang Valley	Migrants, Refugees, Stateless	NGO providing aid, case management, outreach programs, livelihood programs
11	Sarawak	Indigenous Persons, Urban Poor	NGO providing aid
12	Klang Valley	Migrants, Refugees, Stateless, Women	NGO providing aid, case management, outreach programs, livelihood programs
13	Sarawak	Indigenous, Rural Poor, Urban Poor	NGO providing aid
14	Sarawak	Stateless, Urban Poor	NGO providing aid, case managements, legal aid
15	Sarawak	Indigenous Persons, Rural Poor, Women	NGO providing aid, outreach programs, livelihood programs
16	Klang Valley	Refugees	Business/social enterprise employing refugees
17	Klang Valley	Refugees	NGO providing aid, case management, education
18	Sabah	Stateless, Indigenous	NGO providing aid, case management, education
19	Sabah	Stateless, Indigenous	NGO providing aid, case management, education
20	Sabah	Stateless	NGO providing aid, case management, education
21	Klang Valley	Indigenous	NGO providing aid, case management, education
22	Klang Valley	Indigenous	NGO providing aid, case management, education
23	Klang Valley	Stateless	NGO providing aid, case management, education
24	Klang Valley	Indigenous	NGO providing aid
25	Sabah	Stateless, Indigenous	NGO providing aid, outreach programs, education
26	Klang Valley	Stateless	NGO providing aid, outreach programs, education
27	Klang Valley	Refugees, Migrants, Urban Poor	Business employing refugees
28	Klang Valley	Refugees	Individual
29	Penang	Refugees	NGO providing aid, access to medical care
30	Klang Valley	Migrants, Refugees, Stateless	NGO providing aid, access to medical care
31	Sabah	Stateless, Indigenous	NGO providing aid, legal aid, case management
32	Klang Valley	Refugees	NGO providing aid
33	Klang Valley	Indigenous Persons	NGO providing aid, outreach programs, case management
34	Klang Valley	Indigenous Persons	Business providing aid and employment for indigenous persons

### Data collection and analysis

The interviews were conducted either in English, Bahasa Malaysia, Mandarin or combinations of these languages. Five were conducted over the phone and 29 through Zoom. All interviews were recorded with consent (both in audio and visual form, if the participant had turned on their camera for the session) and transcribed verbatim. The transcript was translated into English, if needed. The average duration of the interviews was 60 minutes, with a range between 45 and 75 minutes each. Data collection was led by “N.F.”, our third co-author. ^4^ All interviews were conducted between February 15 ^th^ and March 26 ^th^, 2021. No one else was present in the interviews besides the researcher and participants. The interview question ^5^ was guided by the aim of the study which is to explore how the COVID-19 outbreak and its related policies affects them in health, economic and security aspects, through an ethical lens. The consent form and interview guide can be found as *Extended data* ( Nungsari *et al.,* 2021b).

The transcripts were analysed using HCA ( Bergman, 2010). There are three main steps involved in HCA – the first step (first two sections in Findings) involves sorting and classifying the dataset according to codes. To do this, we conducted a deductive coding thematic analysis using the components of Bandura’s triadic model. The unit of analysis was a question-answer pair, with the full dataset containing 824 excerpts or data points. 541 excerpts were coded as being from the point of view of aid providers, and 283 were coded as being from the perspective of marginalized populations.

The resulting data was coded by three independent coders. To obtain a high and significant level of inter-rater reliability, we followed the coding process developed by MacQueen *et al.* (1998). A codebook was developed based on Bandura’s triadic model, which included intentions, types of agency (two types), type of environment (three types), environmental facilitators and constraints, action potentials (two types) and desired outcomes (achieved and imposed) – resulting in a total of 12 “Bandura themes”, as we describe them.

First, the coders were assigned a small set of non-overlapping transcripts and independently coded them based on the codebook. The 34 transcripts were divided between the three coders almost equally (12:11:11). Then, the coders exchanged transcripts and recoded each other’s work to assess the consistency of code applications. Discussions were conducted to ensure that assessments of the excerpts were done in a similar manner, and repeated until code applications were acceptable and consistent. After each discussion, the coders continued coding more transcripts, and this process was repeated until all transcripts were exhausted. In total, six rounds of discussion (two to three hours each) were conducted to obtain consensus on coding between May 17 ^th^ and July 7 ^th^. The data was coded using Microsoft Excel (version 16.16.27).

The second step (third section in findings), which is conducted through R (version 3.5.1), is visualizing the themes and how they relate to each other using a multidimensional scaling (MDS) graph. MDS addresses “the problem of representing *n* objects geometrically by *n* points, so that the interpoint distances correspond in some sense to experimental dissimilarities between objects” ( Kruskal, 1964). The positions of the 12 Bandura themes relative to each other on our graphs allow us to focus on particularly interesting or surprising correlations.

The third step is taking the MDS findings and recontextualizing them – that is, understanding why relationships exist or why they are the way they are – by using interview data and excerpts.

## Results

The excerpts are divided into those from providers and marginalized populations in order to capture the relationship between agency and environment from both perspectives. It also acknowledges power imbalances in the aid distribution mechanism. The analysis showed that Bandura’s triadic model can account for the aid distribution network during the COVID-19 pandemic in Malaysia. His proposed modes of personal agency were able to capture the agency exercised by both aid providers and marginalized populations, and their relationship with the environment dimensions. The following quote illustrates the analysis process:

… for example, they [aid providers] have 100 packs of food. They will distribute it to us, and we will be distributing it to our community leaders. And that’s most of the things that we do. The challenges with them is that the understanding of each community – the culture, the background, the food, and everything is different from one community to another in Malaysia. [We have] a lot of communities. And then everyone is different from one another. So the NGOs feels that refugees eat their food, [because] their culture is the same. So that is a problem that we get. For example, we eat rice, but other communities may eat roti [bread] or things like that. So the NGO will be buying flour, but we don’t use flour that much, you know, we just use it for dessert. That’s not the main food.

The aid provider demonstrated individual agency by making decisions on the type of aid – i.e., the standardized food packages delivered to marginalized populations. This decision, however, overlooks the cultural diversity of the marginalized populations, which may in turn compromise their individual agency. The lack of cultural understanding represents an imposed environment for the aid providers, which constitutes an example of how environmental constraints lead to negative action potential, and eventually impedes their ability to help effectively because the marginalized populations will not fully utilize this aid.

The quote below demonstrates a better aid distribution mechanism, whereby the providers first consulted the community in need before deciding on the type of aid, hence giving space to marginalized populations to exercise individual agency. This is an environmental facilitator which produces a positive action potential, thus helping to achieve the desired outcome.

During the first phase, it was 100% cash donation, but a lot of them didn’t have a bank account. So it was really difficult to get [transfer] so much money … to one person. And then this one person had to travel to the city to withdraw money and distribute it. So most of them requested for food baskets, and they told us, “okay, this is what we want, we know that it’s probably not something that you agree with, but this is what we eat here, so this is what we want”. So then pretty much our role is just getting the funds, transferring the funds, buying food, and delivering them. What kind of food they want, when to be delivered, most of this is decided by the community themselves. And that’s why different areas are receiving different things.

## Modes of agency for providers and marginalized populations

The modes of agency present among aid providers and marginalized populations, as well as the facilitators and constraints to aid distribution, are summarised in Table 2 and Table 3. Several actors (including the government, healthcare workers, other aid organizations and communities) appear in the context of proxy and collective agency for both these groups. Their appearance highlights the interdependency among different actors in the aid distribution network. As noted by Bandura (1999), people do not live in isolation, but work collectively to accomplish goals. For instance, aid providers have to depend on or work with other aid organizations to overcome capacity constraints in delivering aid packages to remote areas. For the marginalized populations, friends, relatives and community members are crucial in helping them to get by. Many providers and marginalized populations also worked closely with hospitals, clinics and traditional medicine practitioners to distribute healthcare-related aid, including access to COVID-19 testing. Providers were generally able to tap in to the broader networks and resources of more powerful parties, such as the United Nations High Commissioner of Refugees (UNHCR), state and federal governments as well as civil servants. Technology was also a feature of collective agency, where providers often used it to crowdfund specific aid campaigns, disseminate pandemic-related information to marginalized populations or provide awareness campaigns for the general public regarding the underlying social issues surrounding their existence and the provision of aid.

**Table 2.** Modes of agency for providers and marginalized populations.

Individual Agency		Proxy Agency		Collective Agency	
Providers	Marginalized Populations	Providers	Marginalized Populations	Providers	Marginalized Populations
Staff (social workers, teachers, psychologists, cook, management, school principals) Volunteers	Self	Other aid providers Hospitals, clinics, healthcare workers and officials, traditional medicine practitioners United Nations High Commissioner for Refugees (UNHCR) Government Ministry of Education officials Community leaders	Family, friends, community members Hospitals, clinics, healthcare workers and officials, traditional medicine practitioners Aid providers Community leaders Employers/private companies Lawyers	Other aid providers Members and leaders of the marginalized populations Hospitals, clinics, healthcare workers and officials, traditional medicine practitioners Local law enforcement Social media	Family, friends, community members Aid providers Hospitals, clinics, healthcare workers and officials, traditional medicine practitioners

**Table 3.** Environmental facilitators and constraints for aid providers and marginalized populations.

Environmental Facilitators to Aid Distribution		Environmental Constraints to Aid Distribution	
Aid Providers	Marginalized Populations	Aid Providers	Marginalized Populations
*External factors* -Government and political figures who are empathetic and understanding -Support and funding from the government, local citizens and private donors -Technology -Social media -Corporate social responsibility project (CSR) requests from the private sector -Volunteers -Cross-collaboration between providers -Easily accessible COVID-19 information Community leaders from marginalized community *Operations* -Resources and networks to connect marginalized populations to each other in order to form support groups -Ability to navigate the complex environment to get permission to move across districts and states -Established relationships with vulnerable communities -Community and population-mapping -Detailed risk assessments and risk mitigations plans -Online referral mechanisms -Focus on the autonomy and agency of marginalized populations -Pre-existing livelihood programs in vulnerable communities -Sensitization and cultural competency training -Transparency in fundraising	*External factors* -Government and political figures who create empathetic public policies *Social Networks* -Support from family members, friends and community members	*Government & Politics* -Ambiguous and inconsistent government policies -Local law enforcement -Government aid hotlines not functioning -Erratic policymaking -Politicians -Lack of professionalism -Lack of data sharing between aid organizations and the government -Politically motivated aid distribution *Operations* -Lack of funding and donor fatigue -Strict requirements from funders including CSR initiatives from the private sector -Limited mobility -No strong connections to the community -No health protection for volunteers -Insufficient contacts for referrals -Limited capability to serve more communities -Absence of data surrounding vulnerable communities -Lack of empowerment amongst aid organizations -Lack of expertise to address specific issues faced by marginalized populations -Lack of coordination amongst non- governmental organisations (NGOs) *Problems with Marginalized Populations* -Internal politics -Risk of arrest due to increased visibility while receiving aid -The hidden nature of marginalized populations -makes them hard to contact -Fear of spreading COVID-19 to marginalized populations	*Government & Politics* -Unclear government policies cause confusion -Lack of professionalism amongst government officials -Local law enforcement -Government aid hotlines not functioning -Erratic policymaking -Politicians -Sparse and hard-to-access aid from the government *Problems with Providers* -Cultural incompatibilities -Risk of arrest due to increased visibility while receiving aid -Unresponsive of United Nations High Commissioner for Refugees (UNHCR) -Limited or no access to domestic violence support, sexual and reproductive health services *Idiosyncratic problems* -Limited mobility due to roadblocks and poor road infrastructure -No internet infrastructure and spotty cell phone coverage -Distrust in the government and health officials -Lack of legal status -Difficulties filling up paperwork and navigating bureaucracy to access aid -Difficulties and failures in accessing aid from the government and its agencies -Not knowing where to seek help -Digital illiteracy -Xenophobia and discrimination

## Environmental facilitators and environmental constraints to aid distribution

Table 2 summarizes environmental facilitators and constraints in the provision of aid. Environmental facilitators for providers included external factors (e.g., technology and volunteers) and factors related to their own operations (e.g., established relationships with vulnerable communities and transparency in funding). marginalized populations also saw environmental facilitators in the form of external factors (e.g., governments and sympathetic political figures), but also relied heavily on their social networks for access to aid. 

In many cases, environmental facilitators and constraints sprang from the same sources. Providers found that the government and political landscape were a significant source of constraints through issues such as ambiguous and inconsistent government policies, erratic policymaking and a lack of professionalism. Operational constraints include a lack of funding due to donor fatigue, absence of data on vulnerable community needs and a lack of coordination amongst NGOs. They also faced constraints imposed by marginalized populations, which included their internal politics (with some leaders restricting aid to their own family members and friends) and the risk of spreading COVID-19 to marginalized populations, thus limiting aid provision.

Marginalized populations were also constrained by the government and political landscape – they mentioned that government aid was insufficient and hard to access, aid hotlines were not functioning and they frequently got into trouble with local law enforcement, such as when some were arrested or stopped at roadblocks when trying to access aid. Problems with providers included cultural incompatibilities, aid providers such as the UNHCR being unresponsive to requests and calls and having limited or no access to specific resources (e.g., domestic violence support, sexual health and reproductive services). Some constraints were idiosyncratic, pertaining to their vulnerable status, including illiteracy and difficulties in navigating the paperwork and bureaucracy to access aid, xenophobia and discrimination as well as limited mobility due to roadblocks and poor road infrastructure.

## Using multidimensional scaling graphs to depict the relationship between the Bandura themes

**Figure 2.**
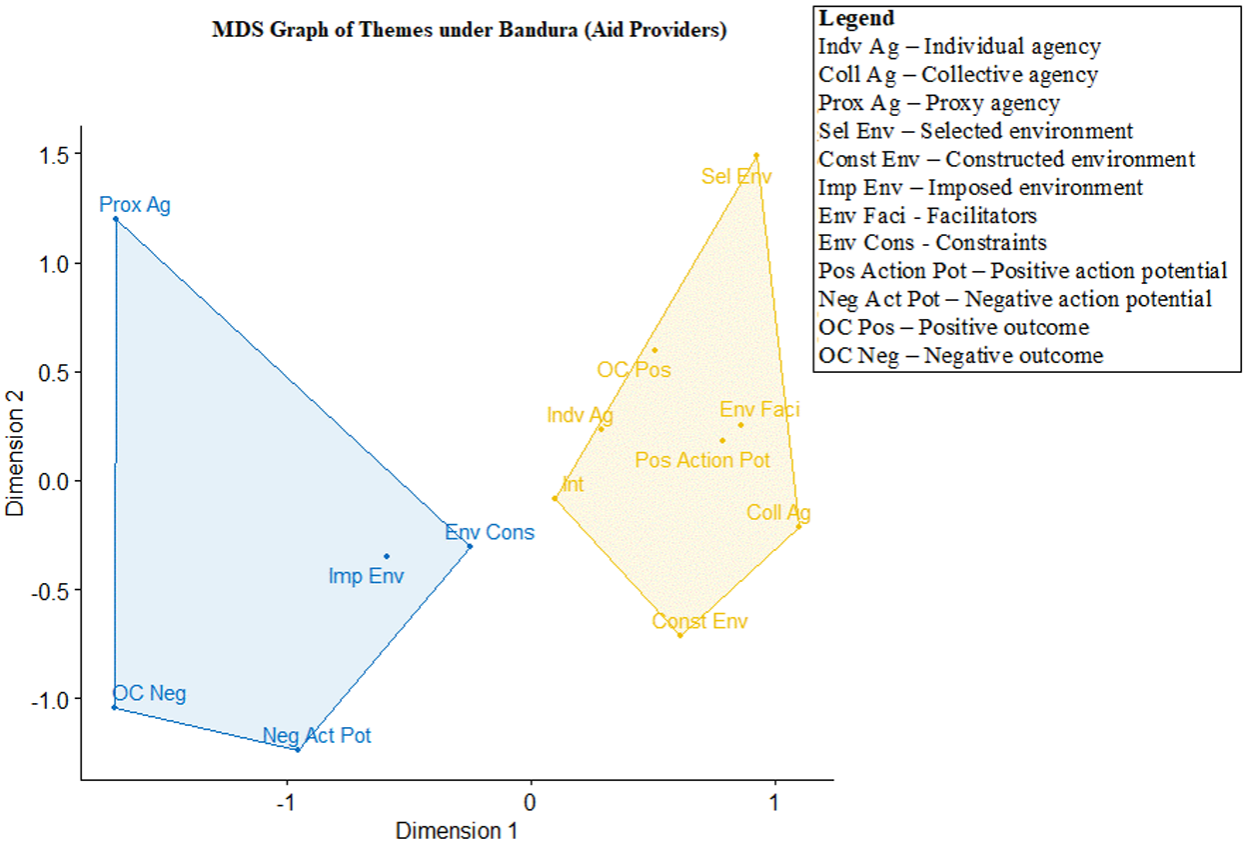
Multidimensional scaling (MDS) of agency themes for aid providers (n=541, stress value=0.105, clustering k=2).

**Figure 3.**
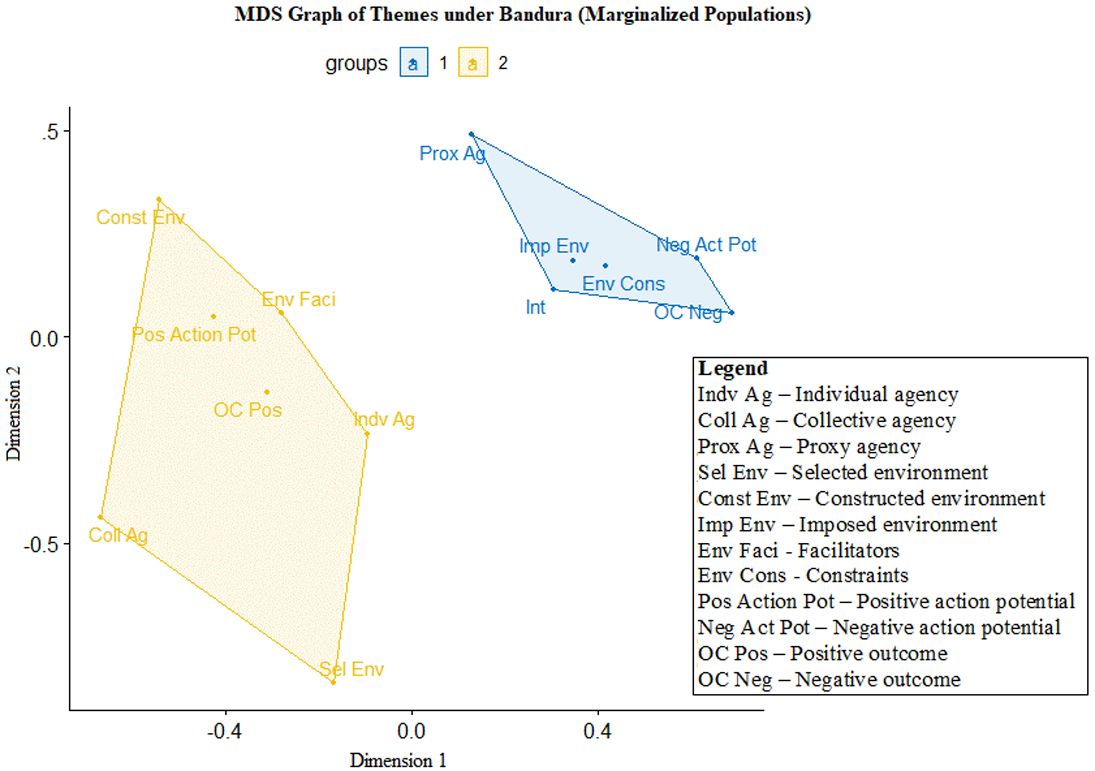
Multidimensional scaling (MDS) of Bandura themes for marginalized populations (n=283, stress value=0.097, clustering k=2).

After the connection between the aid delivery process and Bandura’s triadic model is established, we analysed the co-occurrences of each of the 12 Bandura themes for marginalized populations and aid providers, with the outcomes presented in two separate MDS graphs ( Figure 2 and Figure 3).

Figure 2 shows that two clusters are formed for themes coded among aid providers. The cluster on the left shows an association between proxy agency and all negatively valenced dimensions, including the imposed environment, environment constraints, negative action potential and negative achieved outcomes (i.e., failure to achieve desired goals). This suggests that the reliance on proxy agency by aid providers – namely individuals or organizations *to whom agency was delegated* – often co-occurred with failed outcomes, constants and a negative potential of acting on decision-making. In order to make policy recommendations, we will focus on the difficulties associated with proxy agency in more depth below.

The cluster on the right shows an association between individual and collective agency with other dimensions, including selected environment, constructed environment, environment facilitators, positive action potential and outcomes achieved. This suggests that aid providers were able to exercise agency through individual and collective efforts. They are also able to select the approaches in delivering aid, while the environment enabled positive action potential, eventually enabling them to reach their goals. The link between intentions to help and the cluster on the right indicates that the transition from intention to desired outcomes is better achieved through individual and collective agentive pathways. The stress value for this graph was 0.10, indicating good fit ( Kruskal, 1964).

Figure 3 maps the Bandura themes from the perspective of marginalized populations. The cluster on the right contains the following themes: proxy agency, negative action potential, imposed environment, environmental constraints, outcome not achieved and intentions to survive the pandemic. This is similar to the finding for aid providers – individuals tend to report proxy agency as co-occurring with failed outcomes, the inability to take action and a restrictive, imposed environment with many constraints.

The cluster on the left includes environmental facilitators, individual agency, achieved outcomes, constructed environments, selected environments, positive action potential and collective agency. Thus, individual agency is correlated with outcomes being achieved and the positive potential to act, although marginalized populations sometimes still find themselves in situations where their agency is limited by their environment.

There are two notable facts to note in Figure 2 and Figure 3. The first is that the differences between aid providers and marginalized populations with regard to intention – either the intention of helping marginalized populations (for aid providers) or surviving the pandemic (for marginalized populations). For aid providers, intention is clustered with positively valenced themes (e.g., environmental facilitators, positive action potential and individual agency). For marginalized populations, intention is clustered with negatively valenced themes (e.g., failing to achieve goals, imposed environment, negative action potential and environmental constraints). The second is how proxy agency – i.e., delegating one’s agency to another party – is clustered with negatively valenced themes for both marginalized populations and aid providers. Specifically, proxy agency is often tied to the inability to achieve goals and desired outcomes.

## Recontextualization of the MDS graphs

***Correlations between intention, failure and success.*** We first try to understand why intentions of marginalized populations and providers are clustered on “opposite” ends – specifically, why marginalized populations’ intentions correlate with negative themes and vice versa for providers. In a general sense, these groupings are intuitive – aid providers, by simply existing, were often able to successfully help marginalized populations – and seemingly aided by environmental factors. Marginalized populations, on the other hand, are more numerous and hence, could be expected to be worse-off.

We first focus on aid providers. The key point is that the strength of networks and existing ties are crucial for executing aid programs on the ground. Aid providers were generally able to pull together more resources than marginalized populations to achieve their goals. For example, many were able to leverage the strength of partners in their own networks to distribute coverage.

Our refugee project is all about collaboration because we are we are doing case management and community placement … therefore a lot of our services are tapped from our various NGO partners depending on the type of need. When it comes to medical needs, we will look into Buddhist groups who want to help. When it comes to school, depending on the community, we look at some of the programs by [redacted]. … When it came to the second round of funding, we worked with a local refugee NGO to access the grant … so depends on partners, who is the right partner for the specific community … it’s all about partnership and collaboration for this project.

Smaller grassroots NGOs and aid providers were also able to make decisions fast, bypassing the bureaucracy within large aid organizations or international partners.

… when the MCO hit, that's when we realized that there there's no point of sitting around and planning all of these things because it was so urgent. We just had to go. It was easier for us to adapt to making very fast decisions to having calls to staying up late. And this was a team effort. And I think that's why we are more equipped to deal with the COVID situation, because we were born into it, as opposed to organizations that were that are like a year or two years ahead of us. Their reality and their whole structure would kind of need to change…

Some aid providers who faced a lot of difficulties were also able to self-regulate better due to the strength of their social networks. In the following excerpt (continued from above), the NGO in question set up a process for efficient aid distribution. This was enabled through personal ties with certain powerful individuals. However, it is debatable that this strategy could work for other aid providers (see more in the Discussion).

How the process would work is, we call the community, and then we'd call our team. And then and there, we'd brainstorm. And then from there, we would all tap into our own individual networks and see what strings we can pull. If we need a letter to go someplace, someone would need to “pull strings” with their uncle, their dad or anyone to get us that letter. There was this one time when we needed 60 food packages, and we had to ask for permission to move only a day prior. And we got it, it's because of our own connections.… And that's how COVID just made us realize how privileged each person is with their own connections. You know, everyone has something to offer to the table.

Next, we move on to understanding why marginalized populations’ intentions of surviving were often correlated with negatively valenced themes. One of the main reasons were restrictions on movement. Similar to other developing countries ( Amadasun, 2020), vulnerable groups in Malaysia subsequently experienced increasing human rights violations as well as vulnerabilities. This marginalized person highlighted the difficulties they had regarding roadblocks, which perpetuated their economic livelihood difficulties.

Well, the thing that terrifies me the most after the pandemic happened was going out during lockdown. There was a lot of roadblocks everywhere. After my work permit expired, I didn’t have a visa. So, it was really scary to get out of the house. My biggest fear was actually the police. And it was hard to secure a job, and produce an income. I tried to apply multiples times online for different types of jobs. And I couldn’t get any.

The spillover effects of poor working conditions often translated into increased issues in the healthcare sphere, as highlighted below. Healthcare became more inaccessible, further perpetuating existing problems.

Besides that, not only in terms of healthcare, many of our patients, they are already working in 3D [dangerous, dirty, demeaning] jobs. So what actually happened was with pandemic, issues became exacerbated. And there was an issue in terms of livelihood, a lot of patients could not find work. So yeah, there was an issue we actually saw in our clinic as well as in hospital.

Vulnerable groups were also subject to heightened xenophobia, due to the government targeting foreigners for specific policies, such as raids. Confusing policies also caused a great amount of grief and confusion on the ground. Many marginalized populations found that they could not access aid because of all the uncertainties created by the “flip-flopping” of policymakers:

Financially, it was really difficult, emotionally, I was isolated … not being able to go out, the confusing news we are getting every day. You don’t know what’s gonna happen next or what you should do, because the government didn’t actually make up a plan and stick to it. No, they keep on changing the policies. … Until today, I am terrified about going out.

Marginalized populations also found economic self-empowerment difficult for two main reasons: the first being the economic recession caused by containment measures.

My life is on hold. … I lost my job, and I wasn't able to secure a steady income ever since. I don't have a lot of options. I don't have a lot of choices. I wasn't able to choose for myself. And everything we do now, which is based on what the government is releasing, the information and policies that they are releasing, and it is really confusing. It’s like every day they change their mind … and they are not ashamed. Let's say you made a decision and you are a politician. You made a decision at the end of the day, you're a human being and you were wrong in your decision. But be firm [and] stand by your decision. Don't go out and tell people: “oh, wait, I didn't really mean this. In my announcement, I meant something else, you know.”

The second reason is their lack of citizenship and legal standing, which restricted their possible economic livelihood options.

She arrived to Malaysia two years ago. Then, she overstayed and went to the UN [United Nations], and she tried to apply for it for refugee status. She didn't get anything … except for a small piece of paper that stated her name, passport number, date of birth, and that she came to seek for asylum, but they didn't contact her. … she did contact an NGO telling them that I need help, if you can help me push my file to get to the United Nations. They said they can’t, she has to contact them herself. She has been trying to send emails and phone calls, and apply on the United Nations website. Nothing so far. She had a problem with rent a couple of months ago. And me and another friend, we kind of just collected the money. It was RM600 just to pay her rent. She did approach them [the NGO] that she needs to pay the rent. And the response was: “we’re so sorry, we don’t have enough funds at the moment. There are more urgent cases.” So, they didn’t approach them anymore.

Aid also overlooked the intersectionality of discrimination faced by the individuals and seemed to favour particular groups, such as heads of households (typically men) and discriminated *against* individuals from minority groups *within* the vulnerable population, such as queer individuals. The findings also highlighted the increased burden of household responsibilities on women and girls during the crisis and the lack of access to sexual and reproductive health services for women. This is demonstrated in the following three excerpts:

There were many policies along the way … that were not really right, it was not favouring every group, especially vulnerable groups … [help] was often really towards the household head, and most often this is the male. And because of that, a lot of females are very dependent.
So she's a Syrian, she's a refugee. She's queer. She did approach the same NGO before and no help was offered. I don't know why.
I can say there were changes (for the role of women in the village) for sure. Even for my family itself, like my father…he's like “Oh, actually, a mother is not easy because I have to take care of children.” Usually indigenous families have at least 5 children, that is the minimum. But then during the pandemic, then sometimes the husband will always be out hunting. So during the pandemic, the role of women become even more important, because they have to take care of children, then take care of household chores, and then they need to help their husband in farming, and all those things…the role of women during the pandemic was extremely essential in managing the household. And taking care of children, especially since they weren't able to go to school…teachers sent the children physical homework for those who have no access to the internet. And the women, the moms, gathered a food basket of rice and dishes, and if they have children who go to the same school, then they will gather them together in one place that has connection to the internet and accompany them throughout the lessons to learn from morning until afternoon. Then they also bring packed food for them to eat. I was really impressed and didn't expect this, the role of women and mothers they play in ensuring their children can still learn and have access to education.

Marginalized populations with children also highlighted that surviving means more than simply having enough material resources. The following provider shared an experience of helping a marginalized person with difficulties supervising their children’s schooling for two reasons: the first being inadequate access to technological devices for e-learning, and the second being the lack of knowledge about supervising and helping with their children’s learning. The first can be addressed in aid outreach – the second, however, cannot. This is a systemic issue that requires a more comprehensive solution – such as having dedicated teachers who work with children from such underprivileged backgrounds.

A lot of my clients … they felt very helpless. … Especially those people who have children and right now they have to do e-learning and all these things … there is this person, not a client, but I was helping his daughter. And now he is a carpenter. He has two daughters who need to attend school as well. He couldn't afford all these things. He only has one handphone. He doesn't know what else to do and then the children, even when I get them an iPad, the children are not really interested to study as well. So they all feel like … what do we do in this generation? They all feel a loss as well. … Even with online classes, if the parents are not well equipped to teach their own children, they are not able to actually follow up as well.

Marginalized populations also had difficulties accessing aid and surviving due to their hidden natures – this could be because they are purposefully hiding from local authorities due to their marginalized status in urban settings, or lack physical and technological connectivity to access resources in rural settings. This aid provider highlighted the issues with trying to reach vulnerable groups, with efforts potentially hampered by their lack of phone service and data access.

… they’re actually quite hidden. So I feel during the pandemic, suddenly you can find them because they are reaching out to us and asking for aid. So we found that WhatsApp is a very good way to be able to reach … hidden communities. However, we know more vulnerable communities wouldn't … be able to afford phone credit or a phone. So that's what we were trying to, when we went to the communities. We also asked them to let us know of people who don't have things. … The problem with that is sometimes when we get the list and then we prepare aid for them. We cannot find them. … That’s a limitation that we face. So I think for those who choose to remain hidden, they are hidden … we felt that we were not able to reach the most vulnerable who don't have the means to even have a phone or phone credit.

A number of indigenous persons who were specifically targeted for government assistance were also left behind for this exact reason:

A lot of indigenous persons didn’t manage to get the cash aid … I know that is the most important aid that they need … to pay for things like for the kid’s school and all that. Because they don’t stay in a place where there’s any reception, so they missed the date to register and all that.

Finally, the political dimensions on the ground, such as community leaders or village heads responsible for serving as “intermediaries of aid”, failed to make sure aid was distributed well.

I don't really know the dynamics in the villages. But we heard that the community leaders, they have their own favourites, or they have their own lists of people. So I mean, of course, I'm sure they are good community leaders out there. But we heard, for those who complain that they didn't get the aid, this is what they said to us: they said the community leader didn't include them … they already have a group of people who they want to give the aid to.

***Proxy agency and failed outcomes for marginalized populations.*** As a reminder, proxy agency is exercised when someone gets another party to help achieve their goal. Some parties to whom agency was delegated by marginalized populations in this study include the following (see also Table 2):

- family, friends, and community members;
- hospitals, clinics, healthcare workers and officials and traditional medicine practitioners
- aid organizations;
- community leaders; and
- employers or private companies.

To understand why proxy agency correlates with the failure of marginalized populations to survive, we categorize the qualitative reasons for this failure and intersect them with the party responsible (i.e., those with proxy agency).

The first fact to note is that giving away agency or relying on another party is inherently risky. Survival is then contingent upon somebody else’s agency – i.e., their ability and, more importantly, willingness to help. For example, the following marginalized person depicted his experience trying to find a job in Kuala Lumpur in the midst of the economic downturn:

I walked and asked for jobs. I went to so many places. I couldn’t find any. One of my neighbours is Indian. Mr W. Sometimes when he needs me, he takes me to a job. Like feeding, painting, partition, something like this as a helper for two, three or four days.

In this situation, the marginalized person delegated his agency in terms of obtaining a livelihood to Mr W, who may or may not be able to provide such jobs and employment, particularly when Mr W himself may be affected by the recession. Aid providers also sometimes had to delegate their agency to others, thus creating multiple possible issues.

The issue with sending aid to the island is the transportation … our partner at that time was a local dive centre. At the time, his boatman was in this village that was quarantined, he could not come out. So we couldn't use his boat. Another aid organization then provided us with transportation. We were thankful to them.

Failure to access sources of income due to reliance on intermediaries was commonly repeated in the dataset, as seen in the following excerpts.

Last year during the lockdown, they [my family] were not able to sell their produce outside. Usually there's a middleman that takes the product to sell. This person wasn't allowed in last year. So they were not able to sell.
This organizations collects crafts from the communities they work with and then they will return the profit money. When purchase the crafts, that's their profit money as well. So they didn't manage to do anything like that, to even go to the village and then they didn't participate in any bazaars so they couldn't sell. One of the women we worked with, she basically had zero income from the craft sales itself.

We find that a significant reason for this failure relates directly to lockdowns and restrictions. Marginalized populations often had to rely on a variety of parties – such as the government, UNHCR, NGOs and employers – and many were unable to provide what the marginalized communities needed due to movement and public health restrictions. For example, many programs or resources that were typically provided by the government, such as shelters and in-person training programs, were curtailed.

For our work with the trafficked victims, it was on a total standstill because we could not enter the protection shelter. The government protection shelters closed its doors to outsiders, I think for good reason. But therefore, we couldn’t go in, we couldn’t run services, and so on. We were limited to specialized services … Especially helping them with their testimonies, helping them some of the processes with the deputy prosecutor, and so on…. but in terms of running our usual therapeutic program, our skills-based program and so on – those things had to stop during MCO.

The following aid providers highlighted issues with physically reaching marginalized populations, which was a common problem. Aid providers typically had to absorb any additional costs – in some cases, these added up to hundreds of additional ringgit per trip.

Last month, we planned to go to another city … But we were not able to get a permit from the authorities … because they said the pandemic there wasn’t too serious and we were not encouraged to go. So, we had to stop.
When I was transporting the aid, I was detained by the authorities saying I wasn't allowed to send aid and that I needed a letter from the town council and it needs to be assisted by RELA. ^6^ … But the policy was so that you need to get a new letter for every new day. … This made everything more complicated than necessary. We feel like it's troublesome, sorting out this logistics. And we have to pay for the RELA volunteers, each time we pay them RM50. This was compulsory. And there were some villages we couldn't go in. And we had to leave our aid at designated place that RELA was monitoring … But the problem is the RELA volunteers don't have the data we have about the villages. So sometimes our aid doesn't arrive to the people in need. And the ones who don't have documentation are too afraid to leave the house in fear of getting arrested.

Marginalized populations with intersecting vulnerabilities, such as unaccompanied refugee minors and the elderly, suffered worse from containment measures. For example, the following aid provider highlighted how both the quantity and quality of the usual programs were severely impacted.

Our third area of work was with the refugee minors … we get a lot of unaccompanied separate minors who are in the country. What we do is that we house them in a foster care type of environment, looked after by refugee families from the same ethnicity and background. We incentivize them by supporting the foster family to look after them, rental, food, and things like this. We have a case manager who will take the journey with the kids throughout the case management period … before either they age out or they get resettled or whatever it may be. So, during the pandemic obviously we had to be extra cautious because a lot of the community lives in close proximity. So, we had to be careful for our own case managers to make sure that they don't fall sick. We had to limit their access to meet face to face with the minors during that period, getting a few more things done online. We had to limit access in terms of getting referrals as well. … Our interview and intake process had to change to online. It worked and didn’t work…. you can't really build a rapport enough, you can't to get the child to open up, you can't really see the body language very well…. so you don’t know whether the child is uncomfortable with your questionings. You don’t know whether things are as straightforward as it is because you can’t do a home visit to see whether they’re in a safe environment because you’re obviously seeing them over the screen. You can’t really talk to them … you can’t really develop any gut feelings as to the situation, to see whether the child is vulnerable or not. Whether you need to move the child from that situation due to safety concerns, and so on. We really lose a lot of those things. We had to resort to different ways of providing services.
The elderly also had issues with food and provisions due to the lockdown and had to rely on what little aid they could receive from aid providers. Most of them are already, you know senior citizens, elderly. Normally their children will come during the weekend to send the food supply and all that. But with MCO the children might not be able to just go and send them supply. So it’s quite difficult for them.

Many pre-existing structural issues also exacerbated the difficulties. Restricted access to the internet (which limited access to information about the pandemic and aid relief) and good phone connections proved to be an issue for many vulnerable groups.

Most of them don't have Internet access. The weavers are all like 40s, 50s, and then if they're not highly educated, they don't use a smartphone. Even if they use it's like very minor. You just scroll Facebook. But they do sell things on online as well but they don't have a website because cost to build a website is very expensive, right? Very expensive to have. I don't know if they're on e-commerce websites or not. There’s a microentrepreneur, it's just 20 minutes from where I stay. But once you go there … the internet there was quite bad. … So it's really an issue with connection with basically digital literacy and internet connection stability. So yeah, I mean, to even communicate with them. Unless you've been working with communities for a long time, it's also so difficult to reach them if you don't have this like working relationship … it requires a lot of groundwork for that to happen.

A reliable internet connection was also needed to register for government programs. The fact that many vulnerable groups have been deprived of this need played into their inability to access specifically targeted government aid. An aid provider highlights that although an agency was delegated to help marginalized populations access government support, they simply couldn’t do so because the marginalized populations were either not already in the electronic database, or lacked a good enough internet connection to be put into the database.

… what was sad was that there was about half of the names that we couldn't register especially for indigenous people who live in the interior, because they have never registered for anything before so their info is not with the government.… Some also don’t have ICs [identification cards] or birth certificates. Then it’s more complicated. For villagers who are at the suburbs near the city, it’s not a problem. They can register whenever they want because they have access to the internet. But for those in rural areas, it’s very difficult for them. Sometimes, when we were doing the food aid, during the second lockdown, we focused more of our efforts on helping them register for government assistance. So, there will be cases where the information will be stuck somewhere because the family never registered before or they don’t have any form of identification. So it’s hard because we don’t much except help them to register.

Statelessness was also a core pre-existing condition that prohibited Malaysians who had never registered for formal identification from accessing aid. These include indigenous persons who cannot access formal institutions, such as banking services, due to their lack of formal documents.

Many indigenous people don't have their own bank account. So we had to help with patience and honesty. Those who don't have bank account, we find other people to help them receive the cash. This happened quite a lot.

Aid providers also highlighted specific issues surrounding access to education. This was clearly a persistent problem, as highlighted by an aid provider who ran a learning center catering to minors from vulnerable groups who could not access formal education.

In the Pakatan Harapan government, ^7^ the Education Minister said all stateless students were allowed to go to government schools. But up until now, the amount of stateless students who enter government school is still low.… And we are have visited by education officers who came to us to threaten us … by asking us for documentation from the students and their parents. If we don't show them this documentation, we would be reported … and that violates not just ours, but the community's right to education. And we have never said we are a school, we always say we are an alternative learning center. That is funded and run by the community for the community. So we were not sure whether we would get reported.

With that being said, the following excerpts highlight how the pandemic exacerbated educational access issues. Legitimate concerns surrounded the ability of minors to recover from a severe lack of schooling – studies abroad have noted that “despite favorable conditions, we find that students made little or no progress while learning from home” and that “learning loss was most pronounced among students from disadvantaged homes” ( Engzell *et al.,* 2021).

Our goal here is actually to hire teachers, and then we put the teachers in the refugee learning center, and they will be the ones that craft the syllabus, craft lessons, and then teach the kids as well. So but however, of course, for the past one year … technically, the school can't run. And also at the same time … for Malaysians, or more for like, for B40 communities, there are certain initiatives, they actually give like, free data. Yeah, like free internet access, to help the kids to learn. But also, I understand that in terms of refugee communities … [a] lot of refugee learning center[s] can't really do that. Because most of the kids don't have stable Wi-Fi. At the end of the day, when the lesson ends, they go back to an environment where nobody else can bounce ideas with them. Nobody else can teach them if let's say they don't understand anything. Unlike in a school setting, at least you know, you used to have your friends together with you, you still have like a few teachers that they will come to you and speak to you, converse with you and basically bounce ideas to build a co-learning environment. And I would say these are the two reasons: no internet access and the current situation or environment that they are in is really not conducive for them to go on a virtual learning … so they sort of lost one whole year of class year because of this situation. And of course, currently we are in the midst of building back up again … what we're trying to start [is a] … virtual learning with a small group and trying to put in a lot more [of a] support system for kids and also getting some volunteers to be online mentors. So that at least someone is keeping them in check.
There are a lot of refugee learning centers that have been set up. So because they don't have the formal education, the alternative for the community is they are aware of the importance of education. So they rent a place that in a shop lot, or they will just try to find a place that's close to their community … like a tuition class kind of concept. And they get teachers either from their community or sometimes they will be taken care by like a religious group, churches or NGO. So a lot of time, they actually don't spend money on hiring teachers. However, we all know that … [it] wouldn't be a good school if they don't have good teachers. So in that case, we started because we were all volunteers in the refugee learning center. And we noticed this problem where they are all in a sort of loop, unable to change anything, because the parents don’t have a job to begin with. And hence they cannot earn enough and then send their kids to a better school, things like that. However, the kid seems like they don't really learn as much as well … we realized that one of the main problems is they don't get quality education.

Structural issues – such as lack of legal standing in the country – also caused issues for vulnerable people who were unable to sustain livelihoods.

I tried once to ask them [a multinational aid agency] for help. Like some Arab people here in Malaysia [did, too]. But then I don't think they were really helpful. They tried to give me a job, but after a few weeks, they just fired me because [they said] this is too much responsibilities. You don't have any documents. You don't have a UNHCR card. You don't have anything. Yeah, so that was a problem.

Institutionalized racism directed towards refugees in certain services was also an existing problem that worsened over the pandemic. Many individuals decided not to seek help or were unable to do so due to structural barriers.

I think this is all the trickle-down effects of not legalizing people in the services sector.... There definitely is a lot of discrimination that happens in the hospital between nurses, refugees and doctors … I feel like [this] is a result of the government's inability and competence to deal with it … how is the hospital supposed to deal with refugees that are unable to pay? Refugees that are clearly in need of medical aid, but … are not registered? So, it's conflicting for people in those sectors who are not trained in their six years of medicine, people are not trained to deal with the politics of refugees. So, if I do get frustrated at the hospital, I probably, I just need to remind myself … [this is because of] the government's inability to address all this.

A lack of resources was also a key reason why proxy agency often correlated with failure – those proxies were often constrained in a variety of ways. For example, limited funding restrained many NGOs in aid distribution:

Whatever funds we get is how much we able to help. There are some places we are not able to access. And these places also don't get access to government aid. And when they stop working, a lot of them are labourers, fisherpersons, market sellers or restaurant helpers. So when the sectors are closed, they are not able to go fish or do their work. So their existence is not even acknowledged by the government so they don't have access to aid.

NGOs also suffered from significant capacity constraints, as highlighted by the two excerpts below. In the second excerpt, we see that marginalized populations are unable to access aid from a number of providers.

The biggest challenge was getting funds to run our activities. We are highly dependent on public donations so during MCO lots of sectors were closed so the economy was affected so donors and funders became less.… All our activities depended on money. So imagine our NGO's work is run entirely on the ground, but when we were faced with this pandemic, we were not able to go … face-to-face to the community. But all this was cut off during the pandemic and we had to implement so many different procedures just to enter the village. Some NGOs were not allowed to enter at all to send in items and aid. There was a place where we were meant to drop items off to distribute but we were not sure whether the food we were giving at the designated place got distributed correctly to the people who really needed [it].… A lot of places were blocked by roadblock[s] and even our cars were stopped. So all of this are connected, the lack of funds, restriction on movement, not having jobs, and our activities getting stopped. Everything was not going great for us.
And actually, we received a lot of requests from Facebook. There's this housing area … they reached out to us. At first, we said sorry, we can't help but then because I mentioned that we receive a grant from X. X has a network because they also give a lot of grantees. So X connected us – we are in this WhatsApp group. And then I forwarded their requests … I passed the contact number to the person in charge. And she contacted them. Mostly they need milk, food and pampers for their children. So they give them food. Once or twice but after that, they also decided they also stopped because they say, we also run out of resources. So these people who live in that housing area … they keep messaging us and ask for help.

There were also a significant amount of paternalistic, ethically fraught constraints, where NGOs prevented some marginalized populations from accessing aid. The following aid provider noted that some marginalized populations had very specific basic needs – a request for infant formula was declined because the aid provider wanted to encourage the parents to breastfeed instead.

… when the pandemic hit, we just gave [money]. I don't really like to give direct aid, because I feel it creates that dependence on NGOs, which I feel it can become unhealthy. We always try to empower the community to do things themselves … but during the pandemic … people are hungry. People have children, have no food to eat. So we're like, okay, we just give.… But it's either the funder [who] decided [on what to donate], or we decided based on what when we asked when we asked the community what is needed. We didn't give out baby formula or diapers. There was a lot of requests for baby formula, but I think internationally it's not encouraged. Because sometimes one thing is to encourage breastfeeding. But another thing is also that some babies, they're so used to other things…. [and] they might get sick, or you know … So we didn't give like baby formulas and diapers … but we mostly gave food, food, and then also like soap. And we've also got donations for masks, reusable masks, and like sanitizers, so we encouraged people to practice good hygiene because of the virus. Because some of the funders, they were like, “no, just give food”, but we feel like it's very important to continue to educate, raise awareness on the importance of hygiene during the pandemic.

Marginalized populations who relied on community leaders to help them also faced difficulties in obtaining cash aid. As demonstrated in the literature on development economics, cash aid is often seen as a successful mechanism to address challenges amongst poor and vulnerable populations ( Rawlings & Rubio, 2005), since it can be used in a variety of ways, from buying food to paying loans and rent. However, some aid providers were very uneasy about doing so, thus causing uncomfortable negotiations between themselves and the community leaders.

In one particular community, I made it very clear to this person [a community representative] that the money is to buy the food – just give us a list of people and then give us all the receipts.… [He has] his old method of doing it because in the past, they've been cheated and all these outside people, you see. So, they didn't want to do this. This is somebody that trusts, but his way of trusting is to divide the money, just give you [the community] cash. So I was very angry with them. I said “do not give cash because that is not our cash, to the individual families. You use the cash to buy the food and then you distribute it for them.” But he went ahead and did it. So I was really furious with this particular person, because clearly he was told not to do it. But other than that, he was starting to get angry with the usual. I mean they're supposed to give us photographs and the receipts and so on. And so most of them did, some of them gave, some of them, you know, took a long time to give. But all this kind of normal thing.

Marginalized populations also were unable to access aid through proxy agency because of the politics surrounding their populations or agencies related to their survival. For example, one marginalized person who led a community-based organization pointed out that smaller grassroots organizations tend to get “swallowed up” in the aid distribution landscape.

There is one big NGO in Malaysia, I don't want to say the name. They take advantage of community-based organization like us. So they portray like they are helping us … they try to get funding for us. But they don’t give us the funding. It’s happened many times.... So we as a team learned who to work with and who to not work with. Sometimes community organizations like us have no option. Even if we get 500 (ringgit) to 1,000 to do something, it means a lot, because the work is really important. And then we have to do the work.

Aid was also politicized, with reports of elite capture ( Platteau, 2004) happening on the ground.

Political parties should not be part of the government agencies. You are blurring the line between political parties and the government agencies. And some of these NGOs, they are sort of what we call as government-funded NGOs. So the government actually gives them funding and … they actually use that money to buy things that they then go and distribute. But like I said [I am] pretty sure that some of this money never gets down [to the ground]. They only use part of the money for aid and then maybe they keep 10–20% for themselves. And so I felt that the government should not give money. Should stop this practice of giving allocations for MPs [members of Parliament] for them to give to NGOs … many grassroot societies actually ask for money from the MP and … distribute on behalf of the MPs through the NGOs. I felt that this practice is really a problem, because … Government agencies should have the list of all these people who are in need…. [and] should be the one who are distributing this money. Corruptions definitely happen within these lines of distribution.

Local government agencies were also sometimes at odds with NGOs who worked directly with marginalized populations, causing aid to sometimes be postponed or withheld.

I think after that a lot of NGOs were not allowed to come into the state from Peninsular Malaysia. But I have no verified information on that, it's just what I heard. Before, we felt so helpless. We were only communicating with a local disaster response team – they were the middle person. And actually, there's just a miscommunication, which is why I'm not sure how the coordination can be done at the national level, because at this very small level, there were already misunderstanding … I didn't try to reach out to the district health office.… the local disaster response team was … afraid to share contacts from the district health office with us, and we didn’t know how to reach the correct person. When our situation went viral, the district health office came to us…. The good thing is that we have a very good relationship now with the health office. The bad thing is that we lose our relationship with the local disaster response team.

Due to the large number of deportations and arrests at the beginning of the pandemic, undocumented communities have lost a significant amount of trust in the government. This was clearly a point of trauma for many marginalized populations – the trust which they extended to healthcare workers at the start of the pandemic was clearly betrayed, leading to an inability to access healthcare services and vaccines.

Recently, they put out a statement saying that migrants and refugees can now take the vaccine. But that's a fear on our side, because the last time they did it, they arrested a bunch of them, even though they are recognized.
Even my two students have faced police abuse that was terrible, their entire family experienced it. So these kind of stories, wrongfully detained, abuse of power, this is what caused, you know, the stifling of voices to build themselves up. But it's very easy to bully them, and scapegoating the stateless communities by blaming them, saying they brought over the pandemic.... these become traumatic experiences for the stateless community, into their psyche. So it's not like they don't want to work together with authorities. But because of all the many previous cases of abuse, this is what happens. So when health officers come by for screenings, they will run away. It's very difficult for them to work together. So another thing we did was a health campaign. We always brought the latest COVID-19 information to them through daily announcements, how many cases, COVID-19 precautions they can take, and requests for them to work with them [the health authorities] if they are ill so they can send them to hospital…. But we were not successful in convincing those who were COVID-19 positive in going to the quarantine center [on the mainland]. We managed to convince them to get tested but not to go to the quarantine center. So that's what we did in terms of health.

Many marginalized populations were also unable to access aid through their proxies due to incompetency on the part of the government – both cultural and operational. Cultural incompetency was primarily due to the fact that aid providers incorrectly assumed the marginalized populations had specific needs, and often provided help that ignored or negatively impacted their agency. We saw an excerpt earlier where refugees were not given culture-specific foods. But this issue also extended to indigenous populations.

And during that time a lot of people did help, NGO, government, even JAKOA [the Department of Orang Asli Development] also helped. But the help didn't really encompass what the indigenous people needed. Each village won't try to deny that they did receive help and all. But what we request for doesn't always seem to get to us. We only get rice, oil, sugar. And the rest of our needs … are not met…. So what do they [the Orang Asli] need? Baby formula, onion etc. So it's like they [the authorities] only give whatever they feel like giving only, and not what the OA wants. Families who need baby formula, it's very hard for them to get it because they can't even go out to buy any. So when they have no income and the economy is shut so your farming also is not an option, then families will come to us begging for help, saying they already have food and that they have already previously requested these items but some of the people providing aid will say, we already gave them food, [there is] no need to give them extra things. When in reality, it's not extra things, it's things they need for their baby or children to survive on…. The parents themselves are willing to be hungry … They don't even know what's happening with MCO or COVID-19. So regardless of whether it's NGO or government aid, you need to ask the family what they need. Each family has different needs.

Government agencies, however, were also operationally incompetent. Many marginalized populations reported being unable to access government resources and services, such as reaching their hotlines.

[The government] has been a bit slower to respond … during the MCO time when we are not allowed to help, we got informed that a lot of people called us and they need help.… most of the time when we were helping people to call [the government hotlines], they did not answer … what should we do? We’re not sure if it is a lack of manpower or because they’re really not working.

Marginalized populations were also placed in undignified positions by aid providers, who cared more about marketing their aid distribution. As seen in the excerpts below, they also sometimes put up with certain indignities (such as being the subjects of photographs) to access aid.

When you do charity work and you take money in our name, and then you give it to someone else because you don't want to have a bad name and things like that, it’s really, really not nice. It’s not even human.… but some people also tried to be really nice in the lockdown. They tried to bring food packets, they asked us to go and distribute, they came with us to distribute, they take pictures, but they don’t understand the needs of the people. But we needed to help people, but it became too much for us. It’s like we became a platform to fulfil their wishes…. they don’t treat us as a platform where we build community. They tried to listen to themselves and they follow their rules but not ours, you know?
A lot of [vulnerable] communities just accept the help [from NGOs]. So it's a bit hard. So okay if they [the NGOs] want to take picture[s] … we, the indigenous people, can't control what they do with the picture, like where they post and what they are saying about us. We have to just trust them. So, we kind of already given up with these things … it’s become normal to them already.

Finally, we would like to highlight the most problematic and troubling example of how proxy agency can lead to failed outcomes. Marginalized populations and aid providers have reported members of vulnerable groups going missing or becoming highly uncontactable after being arrested. In the past, vulnerable communities in Malaysia have relied on the UNHCR to free their loved ones (who actually held UNHCR cards) from detention centres and jails, but this practice was halted during different stages of the pandemic. In a news article dated April 1 ^st^, 2021, UNHCR Malaysia stated that:

UNHCR seeks access to immigration detention centres so that we can help separate refugees from undocumented immigrants. Once we have determined who is in need of international protection and should not be returned, authorities can proceed with immigration formalities for undocumented immigrants, while remaining confident that Malaysia is upholding international human rights principles with respect to refugees and asylum seekers. ( UNHCR Malaysia, 2021)

With the UNHCR no longer being allowed to perform this function, many marginalized persons have reported anguish and panic when they were not able to trace their loved ones who moved through the detention system.

During the raids, not many of my community members were arrested, but there are some of them get arrested because of breaking or violating the MCO law…. It took some time … like two to three months [of] being detained. Yeah, and you won't believe it. There's one guy, our community member, he went out after 11. And then he has a UN card, but he was arrested and our community members tried his [their] best to get the person released. But it took, I think, one month, or more than one month. He was at the court. We had to communicate with him to get assistance from UNHCR. But then finally he was released because when he was arrested, his wife was with one-and-a-half-month-old baby. So, the wife was really very worried. And we couldn't find [him] anywhere because he was taken away by police car, but [we] don't know where he was taken to. So, it took us quite a while to find out where he was being detained.

Delays in receiving information also prevented aid providers from stepping in at various points during detention, causing grief to the family and friends of the detainees.

Maybe UNHCR has been denied access to detentions and prisons for a long time, almost going to be two years. So, it puts us in a really hard situation where it’s very difficult to find out if someone gets arrested. From the point they get arrested, we totally lose contact with them. We don’t know when is their court procedures, and we try to communicate, or we always ask our community members to inform as much information they have when one of them gets arrested. So, through some friends or family members or some relatives, we will get information and start communicating with UNHCR and NGOs that can actually represent them. Most of the time, it’s not effective, because by the time we know where they are, it’s already too late. They are done and past all of the court procedures, they’ve been sentenced … they’re about to be transferred to detention centres, and it’s really very difficult for us to help from that stage. The information is already very delayed.

Aid providers also reported that undocumented individuals who could not prove their refugee status were forced to accept deportation because they either did not understand the process, or could not stand detention center conditions. The aid providers noted that since they were not able to reach said individuals, they were not sure whether those individuals understood the realities outside when they requested to be deported.

We understand that many detainees become deportees because they couldn't stand the detention conditions anymore … some of them were being kept away for many years, lost contact with their family members … there are groups that agree that because they've suffered for a long time, they want to go back. And we have to respect that…. At the same time, we feel worry and concerned because there's no Wi-Fi in the detention. So, we're not sure whether they understand or [if] they know about the current situation in the country … the only questions we all have is why now? Why in this situation? What is the intention behind [it]? And what are the agreements that created this whole process?

**Figure 4.**
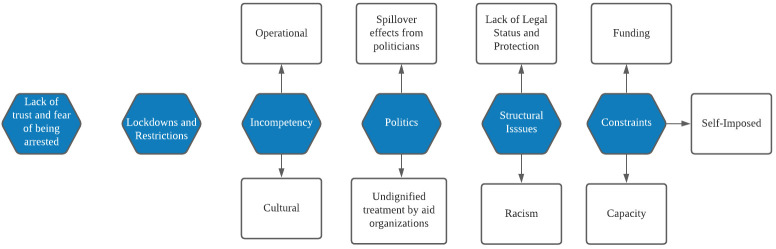
A summary of why proxy agency for marginalized populations correlated with failed outcomes and negatively valenced themes.

In summary, we see that the reasons why proxy agency was correlated with failed outcomes are diverse – these include structural issues relating to the status of the marginalized populations, lockdowns and restrictions imposed due to the pandemic, racism and incompetency (both cultural and operational). We summarize our findings in Figure 4.

## Discussion

In this study, we identified all modes of agency for both aid providers and marginalized populations according to Bandura’s triadic model. Specifically, we identified (1) parties to which agency had been delegated by providers and marginalized populations (i.e., proxy agency) and (2) those who worked together with providers and marginalized populations to achieve their two main goals: delivering aid to marginalized populations and surviving the pandemic respectively. The analysis of the modes of agency revealed that agency was derived from a diverse range of external parties, which included employers and private companies, healthcare workers, local law enforcement, social media and the government.

Using MDS and recontextualization, we then mapped out the environmental facilitators and constraints which prevented providers and marginalized populations from exercising agency over the course of the pandemic to understand factors that either helped alleviate or caused more suffering. We found that the intentions of aid providers, which are to help vulnerable populations often correlated with success in achieving outcomes, whereas the intentions of marginalized populations, which are to survive during the pandemic often correlated with failure. We found that resources – both social and material – played a huge role in this respect. However, proxy agency was often problematic for marginalized populations, and relying on this mode of agency often resulted in failed outcomes. We identified the main reasons for this, as summarized in Figure 4: a lack of trust and fear of being arrested; lockdowns and restrictions; incompetence; politics; structural issues; and constraints (i.e., funding, capacity and self-imposed).

Once we have performed agency landscape mapping, what do we do with this information? How can we suggest policies pertaining to the distribution of aid in order to maximize the total number of marginalized populations who are able to successful access resources, and thus survive the pandemic?

Based on the information in Table 3, our first of three policy recommendations is as follows. It is clear that although some environmental constraints are the direct results of the pandemic (such as lack of or limited mobility), many others were not, and these should be removed from the landscape to improve agency. For example, eliminating erratic policymaking by government agencies and poor communication which disrupted access to and distribution of aid should be changed in order to improve coordination on the ground. Aid organizations reported failing to conduct extensive community mapping or establish relationships with known vulnerable communities, whom they were unable to reach out to due to the lack of trust and existing structures. We recommend, again, that for all NGOs and organizations working with vulnerable communities, relationship-building is crucial. The aid distribution and provision must be participatory and inclusive for the communities involved. The government also needs to step up during crises and improve outreach to distribute aid as widely as possible through rights-based and people-centred approach. Intra-government agencies collaboration and planning are essential to ensure consistent and effective policies in containing the COVID-19 outbreak. Policies like immigration raids and mass detention, for instance, have proven to be having adverse impacts on vulnerable communities as well as jeopardizing the public health policies. But as we saw in Table 3, marginalized populations were often constrained in terms of agency by a lack of professionalism amongst government officials, outreach methods such as hotlines were not working and law enforcement agencies behaved aggressively. Aid providers also faced a variety of government constraints, which included a lack of data-sharing between aid organizations and local agencies, creating silos which inhibited aid providers from reaching out to help. In many cases, government agencies’ ultimate goals were to help vulnerable groups, but in practice, they were unsuccessful because execution was so overwhelmingly haphazard and uncoordinated.

The second policy recommendation relates to our finding that proxy agency is problematic for marginalized populations. In Figure 4, we summarise how a reliance on other parties can be incredibly problematic for marginalized populations – especially when proxy agency is multi-tiered (i.e., proxy agents themselves often rely on other proxy agents). Although the reasons are diverse, we see that such agency is clearly a constraint on marginalized populations. With that being said, community resilience needs to be enhanced to reduce the need for proxy agents. Reliance on external parties, although necessary at times, reduces marginalized populations’ personal agency in making choices and decisions as they see fit, weakens their ability to respond to crises for their own good and causes them to suffer when their proxy agents fail to deliver. Hence, we recommend that the government and aid providers invest in creating self-reliance programs for vulnerable communities – these could include more extensive livelihood programs, direct capacity building programs, or encouraging and funding existing self-sufficiency programs (e.g., enhancing farming programs in rural areas for the appropriate communities). These vulnerable groups will have a better chance of self-sustaining themselves in future crises. However, we stress that this does not mean that the government and multinational aid and humanitarian organizations should wash their hands of this issue completely – rather, that significantly more resources should be invested into vulnerable communities, especially since so many structural barriers currently exist.

The final policy recommendation is on the role of international aid organizations, with regard to specific vulnerable groups. We saw in the Results section that the UNHCR’s failure to extract detainees with UNHCR cards meant that the “agency chain” between detainees and their loved ones completely broke. Sometimes, this even ended with their deportation, often because detainees completely lost all agency, unaware of current happenings outside the jail and in their home country. ^8^ International aid organizations typically take responsibility for vulnerable groups when their issues are not covered by the local federal governments and agencies – this includes, for example, dealing with detained refugees. With this being the case, the removal of such a large and very important proxy within the agency landscape means that huge parts of vulnerable networks will suffer. Thus, we strongly urge the government to respect the principle of non-refoulement under its international human rights obligations and to allow continued activities and existing arrangements with international organizations to take place – even during the pandemic, as long as the required restrictions and parameters are observed.

## Strengths and limitations

Using an approach that combines the consequentialist ethical framework and public health ethical principles, this study is one of the few studies that explores the agency of marginalised communities and aid providers during the COVID-19 pandemic through an ethical lens. However, this study also has several limitations. First, as the study is centred on the issues faced by vulnerable communities, the findings may not be applicable to other population. Second, responses by policymakers and grassroots organisations towards the pandemic vary by country. Hence, generalisation of these findings in other settings must be done with caution. Third, we also recognise that this qualitative study is of relatively small scale and are not representative of all populations. However, through purposive sampling, we had included participants of diverse background and social identities. We also believe that the triangulation of findings through desk review and interviews with multiple stakeholders provides a comprehensive understanding on the impacts of pandemic from different perspectives.

## Conclusion

This study comprehensively mapped the “agency landscape” of vulnerable groups and aid organizations in Malaysia during the first few months of the COVID-19 pandemic. It classified the modes of agency for marginalized populations and aid providers, highlighting the environmental facilitators of and constraints for each of these groups in achieving their goals. These include simply surviving and obtaining help (for marginalized populations) and successfully providing aid (for aid providers). Using HCA, we explored the relationship between each component of the agency landscape to understand the relationships between them. We found that marginalized populations’ intentions of surviving often rely on external actors as proxy and correlate with failed outcomes, and aid providers’ intentions of disseminating aid typically correlate with successful outcomes through individual agency and collective agency. Additionally, we found that the use of proxy agency is problematic for marginalized populations and determine why that is the case. Finally, the paper ended with the presentation of three policy recommendations which prioritise marginalized populations and their needs, while removing barriers to accessing aid.

## Data availability

### Underlying data

The transcripts underlying the paper are stored in a confidential, secure location due to the sensitive nature of its contents. Most participants in this study were either undocumented or worked closely with undocumented people, who are often at odds with the local law enforcement and are at a heightened risk for arrests, abuse, and deportations. We are willing to share the transcripts in full with anyone who sends us an email request, but in order to protect anonymity and the confidentiality of our participants, cannot store it in an open data repository. Any individuals who are interested in requesting access to the dataset can do so by explaining the reasons why in an email to the corresponding author to the study, M.N. at her email address: melati@mit.edu. Permission will be granted on a case-by-case basis, and data will be anonymized by removing any identifying information of participants before provided.

### Extended data

Open Science Framework: Exploring Ethical Challenges Faced during the COVID-19 Pandemic in Malaysia. http://doi.org/10.17605/OSF.IO/YJ9AF ( Nungsari *et al.,* 2021b).

This project contains the following extended data:

- ECCO Consent forms.pdf (consent forms for participants which are divided into six categories: employer, NGO, indigenous person, migrant worker, refugee, undocumented person)
- ECCO study instrument.pdf (interview guide)

Data are available under the terms of the Creative Commons Zero "No rights reserved" data waiver (CC0 1.0 Public domain dedication).

## Notes

^1^ Melati is an Assistant Professor of Economics at Asia School of Business (ASB) and a Research Affiliate at MIT Sloan School of Management. Hui Yin is a Senior Research Associate at ASB. Nicole Fong is a Research Affiliate at ASB. Veena is the Founder of Diode Consultancy. Melati is the corresponding author. Email: melati@mit.edu. Address: Asia School of Business in Collaboration with MIT Sloan School of Management, 11 Jalan Dato’ Onn, Kuala Lumpur, 50480, Malaysia. Number: +6013-290-3225. 

^2^ ECS-MP approved by the Head of Research Integrity and Ethics Unit, Universiti Malaya. WHO ERC approval with Protocol ID number: CERC.0075.

^3^ 41 of the individuals who declined either did not respond to the call for participation or had scheduling conflicts. Five did not feel like they had the right expertise or had already participated in similar studies beforehand. Two gave no reason, perhaps this was because the data was collected during the lockdown and many organisations and individuals were still providing food aid to the communities and working on the ground.

^4^ N.F. is a researcher-activist, cis-woman and trained economist with three years of research and human rights advocacy experience.

^5^ The interview guide is available in Extended Data below.

^6^ RELA (Jabatan Sukarelawan Malaysia) is the Malaysian Volunteer Corps Department, a civil volunteer corps formed by the Malaysian government in 1972.

^7^ It was in power from May 2018 until March 2020, replacing the previously untoppled Barisan Nasional.

^8^ In February 2021, the Malaysian government deported more than 1,000 detainees to Myanmar at the beginning of its violent military coup. The Immigration Department insisted that refugees and asylum-seekers were not part of this group ( *Al Jazeera and News Agencies*, 2021).
