# Supplementary material for: An agency mapping of marginalized communities and aid providers during the COVID-19 pandemic in Malaysia
Source: Wellcome Open Res. 2022 Jan 20;6:323. Originally published 2021 Nov 25. [Version 2] doi: 10.12688/wellcomeopenres.17315.2 (PMC11736104; doi:10.12688/wellcomeopenres.17315.2)
Supplement: Peer review history

# Reviewer report: Reviewer response for version 2

**DOI:** 10.21956/wellcomeopenres.19470.r116210

**Published:** 2025-01-15

**Authors:** Christopher Phillipson

**Affiliations:** 1 The University of Manchester, Manchester, England, UK

- This is an interesting review of the landscape concerned with providing aid to marginalized populations, with novel use of Bandura's social cognitive theory applied to the impact of COVID-19 on vulnerable populations in Malaysia. There is a genuine research problem here concerning the interaction between NGOs, their funders, and the grassroots organizations to which they relate: hence the potential of the article. There is limited literature of this kind - especially regarding that dealing with marginalized populations.
- The findings from the research certainly confirm equivalent work in the West: namely, failure to address the needs of migrants and refugees; difficulties of reaching indigenous populations; lack of trust in, as well as incompetence on the part of, government; and failure to understand cultural differences.
- These and other issues were highlighted in the findings and make an important contribution to the literature. There is though a difficulty with the paper which does need to be addressed, namely, the balance in the sample between NGOs and marginalized individuals. Essentially, the study reports on the work of 31 NGOs: their relationships with their funders and the communities they serve. The data provided was certainly interesting and relevant to researchers and policy-makers. Against this, there  is insufficient material to make claims about the views of marginalized populations - the three individuals who were recruited do not give scope for this. My solution would be to focus the article on the work of the aid providers alone, still maintaining the original theoretical and methodological approach. This would not exclude of course discussion of 'marginalized populations' and their relationship to the aid organizations; it would though preclude material which claimed to speak on their behalf - that does require rather more data. 

Is the work clearly and accurately presented and does it cite the current literature?

Yes

If applicable, is the statistical analysis and its interpretation appropriate?

Yes

Are all the source data underlying the results available to ensure full reproducibility?

Partly

Is the study design appropriate and is the work technically sound?

Partly

Are the conclusions drawn adequately supported by the results?

Yes

Are sufficient details of methods and analysis provided to allow replication by others?

Partly

Reviewer Expertise:

Community studies

I confirm that I have read this submission and believe that I have an appropriate level of expertise to confirm that it is of an acceptable scientific standard, however I have significant reservations, as outlined above.

# Reviewer report: Reviewer response for version 2

**DOI:** 10.21956/wellcomeopenres.19470.r88239

**Published:** 2024-09-02

**Authors:** Prince Agwu

**Affiliations:** 1 University of Dundee School of Humanities Social Sciences and Law (Ringgold ID: 150731), Dundee, Scotland, UK

Great study but with deep presentation issues. I have provided comments to help fix these concerns.

 The background in the abstract gives the impression of your focus being the ‘agency’ of CSOs. At the same time, the topic says it is the ‘agency’ of marginalised communities you are concerned about. It would help if you resolve this conflict. You should try to ensure that the problem of the study is clear in the background of your abstract.

 The line ‘civil society organizations (CSOs) and members’ in your abstract is confusing. It comes to the reader that both are different.

 Your abstract seems unclear because the ‘agency’ focus is equally unclear. If you are focused on both agencies, i.e., service providers and users, you should also be clear, and importantly, conceptualise the problem well around them.

 Of the 34 participants, state numbers for providers and users.

 Your results in the abstract is not appealing to wider readership. Your abstract is the eye into your article and should be readership-friendly. I wonder why the results section in the abstract is too tiny compared to your methods, especially when your article doesn’t set out to discuss methods.

 Arrange your keywords alphabetically and six keywords should be ideal.

 There is a difference between doctor and medical doctor. Also, ‘forcing doctors to ‘play God’…’ isn’t a great academic expression. Besides, medical doctors weren’t acting alone – we had other health workers. Try not to describe the health setting in a way that advances the parochial narrative of ‘medical doctor-dominated’.

 ‘They **are** disproportionately impacted …’ – do you mean ‘were’.

 I have read your first three paragraphs in the introduction, and I can’t see ‘why the study’.  The hook of your article has to be located in the early paragraphs. That’s what readers want to see. You missed the same in your article. In very simple expressions ensure that the hook of your piece is positioned in your earlier narratives and try to stick further arguments around it.

 Somewhere in your introduction, you should clarify what you mean by ‘human agency and power imbalances’. We should be writing for everyone and not for experts alone.

 Your theoretical background is expected to swing like a pendulum, touching both the agency of service users and providers and bringing both to the contextual perspective in a way that anyone can understand the argument. Currently, it seems one-sided. The four decomposed agentive processes should reflect both users and providers and should highlight the power issue you had earlier raised.

 Figure 1 misses out CSOs and does not capture ‘power imbalances’ as earlier argued. Please, revise. This is why I have suggested to get the arguments right from the onset.

 Since the theory informed the code book, you should say how it informed the questions as well, especially since you are using deductive coding.

 It will be interesting to see how ‘R’ helped with the analysis. I appreciate what the authors have provided, but more information will make sense.

 Meanwhile, the authors should look up Consolidated Criteria for Reporting Qualitative Research (COREQ) - https://www.equator-network.org/reporting-guidelines/coreq/, and ensure they are guided properly by the 32-item checklist. State when the study was conducted.

 What about those you never got a reply from? You should state the number too in footnote 3. Meanwhile, it is not acceptable to start a sentence with number – instead of ’41 of the individual who declined …’, you should state Forty-one…

 Somewhere in your methods, you should state your themes and ensure they are aligned with the research questions or objectives of study which should be mentioned in the last paragraph of your introduction. Those would show what the research gaps are, and what we are comparing the results against.

 Under your results, the first section has no theme – why?

 You really do need to clarify the ‘agency’ concept in your article and do this early enough. The theme ‘modes of agency …’, conflicts with the idea of agency as ‘choice… decision-making capacity’. The arguments have always been about agency versus structure, and somehow this plays out at the proxy and collective levels but is situated as agency and not structure. These are some articles that have argued out the concept of ‘agency’ and you may want to see how you adapt to your arguments - https://journals.sagepub.com/doi/full/10.1177/00380385211020231 and https://bristoluniversitypressdigital.com/view/journals/frs/13/3/article-p442.xml

 Your results section seems to be structured more like an introduction or discussion, which is unusual for qualitative research. In qualitative studies, it's crucial to present illustrative quotes that support and elaborate on the subjects of your themes

 The presentation of results under the ‘recontextualization of the MDS graphs’ and ‘proxy agency and failed outcomes’ is overly long. Please, cut down to the most meaningful discourses. Also, it is wrong to have presented the ‘R’ analysis of the data using figures without augmenting them with illustrative quotes. This has to be done in one breath.

 Arguments around politics, government, etc., are structure-inclined and not agency. Again, clarify these concepts. And I wonder how they are included in the mix of community agency versus CSOs agency.

 Your results are not well organised to be punchy and I do not see them addressing specific study objectives. I have provided how you can correct this in prior comments.

 This study is simply saying that there is a top-bottom relationship between CSOs and communities, especially vulnerable (note that this is different from marginalised – because you have to prove why, how, and who is marginalizing them), and this is happening because of the uneven spread in decision-making. So, the question is how do we correct this imbalance by democratizing determinants of community needs and meeting the needs as well? I do not expect this argument to be complex. I am urging the authors to present the findings from this study more concisely and coherently.

Is the work clearly and accurately presented and does it cite the current literature?

Partly

If applicable, is the statistical analysis and its interpretation appropriate?

Not applicable

Are all the source data underlying the results available to ensure full reproducibility?

Partly

Is the study design appropriate and is the work technically sound?

Yes

Are the conclusions drawn adequately supported by the results?

No

Are sufficient details of methods and analysis provided to allow replication by others?

Partly

Reviewer Expertise:

Social Policy in Health Systems and Migration

I confirm that I have read this submission and believe that I have an appropriate level of expertise to confirm that it is of an acceptable scientific standard, however I have significant reservations, as outlined above.

# Reviewer report: Reviewer response for version 1

**DOI:** 10.21956/wellcomeopenres.19143.r47259

**Published:** 2021-12-08

**Authors:** Zinette Bergman

**Affiliations:** 1 Mixed Methods Program, University of Michigan, Ann Arbor, MI, USA; 2 Social Research and Methodology, Department of Social Sciences, University of Basel, Basel, Switzerland

Dear Authors,

 Thank you for this interesting and timely research. I hope my comments below are helpful in the final revision of your manuscript. Overall, the article is well-written, the methods sophisticated, the theoretical framework, methods, and findings clearly communicated, and the internal logic sound and easily understood. The justification for the research as outlined on pg. 3 creates an excellent tension for the importance of the research, and you make very good use of figures and tables to guide your reader throughout. Many of the sections are crisp and to the point. Thank you for your scholarly contribution!

**From a methodological perspective, I have two (small) recommendations:**

- First, the idea of data saturation in qualitative research belongs to a very specific approach in Grounded Theory. Applying it outside of the methodological frame of theoretical sampling seems attractive since it mimics the logic of representivity (i.e. we stopped conducting interviews when data saturation was reached, which implicitly suggests that all themes/characteristics have been uncovered), but of course, in the context of non-probabilistic sampling, it is entirely possible (even very likely) that sampling 34 *different* people could yield a different set of characteristics, which in turn would lead to a different 'data saturation' point and outcome. The point here is that 'data saturation' is the process through which multiple stages of theory development have been refined through multiple site visits and stages of analysis, falsification, and (re)development. It refers to an analytic process not to sampling. Applying it to denote a sampling strategy (even though done in some QL literature) is a misapplication of the concept and, given the sophistication of the methods you employ here, I would avoid making this mistake.
- Second, a similar problem arises when you use the term 'correlation', which belongs to a specific set of methods and connects to a very specific language and scientific logic. In dimensional analysis, especially based on a dissimilarity matrix configuration, the relationship between themes are co-occurrences and it is more appropriate to discuss them in terms of how they are linked or associated with each other (but not correlated). I would avoid using the term correlation in an HCA framework.

**In terms of presenting your findings:**

- A three-step analysis can be difficult for readers to hold in their heads while following the line of argumentation. I would recommend inserting summary sections at the end of important parts to highlight to your reader what the main points were before they move on to the next section. While reading the manuscript, for example, I would have found a summary section helpful on pages 8 (before the section on 'Using MDS graphs') and 14 (before proxy agency).
- The final analytic section starting on page 14 still needs some work. It is very dense and can seem list-like. While the data excerpts provide wonderful insight, it is sometimes hard to follow how the many diverse issues you introduce here come together. Overusing 'also' (it appears 17 times in this section) is an indication of the list-like nature of this part of the manuscript. Figure 4 seems to provide a good conceptual overview of these divergent issues. I wonder if you could think of a way to restructure (maybe with subheadings, a stronger thematic logic that explicitly groups different issues together, or aligning more explicitly with Figure 4) this section to make it less list-like and bring out the analytic logic of your findings. I want to stress here that my comment is not related to the content, but rather to how it is being presented.
- Finally, the inclusion of the news article on the UNHCR on pg. 19 undermines the systematicity of your empirical analysis. I would remove this anecdotal reference. I think your overall point will hold with the data excerpt you provide after that. 

**In the discussion section:**

- It is not always clear how your recommendations are an outcome of your findings. If you strategically use summary sections earlier on in the manuscript, this would be a good time to return to them to highlight more clearly how your recommendations explicitly connect to and build from your findings presented earlier.

**In the strengths and limitation section:**

- The first sentence starts with "Using an approach that combines the consequentialist ethical framework and public health ethical principles..." seems out of place since this is the first time you are referring to this.

**General comments:**

- In both Figures 2 & 3, 'Int - Intentions', is missing from the legend.
- The paper could do with a final rework to remove minor spelling and grammar errors. For example, "...lack of understanding of how is human agency exercised" (pg. 1), "...not only for creating an effective and inclusive aid delivery mechanism s" (pg. 1).

Again, excellent research. Thank you for your contribution and good luck with your final revisions.

Is the work clearly and accurately presented and does it cite the current literature?

Yes

If applicable, is the statistical analysis and its interpretation appropriate?

Yes

Are all the source data underlying the results available to ensure full reproducibility?

Yes

Is the study design appropriate and is the work technically sound?

Yes

Are the conclusions drawn adequately supported by the results?

Yes

Are sufficient details of methods and analysis provided to allow replication by others?

Yes

Reviewer Expertise:

Content Configuration Analysis, Hermeneutic Content Analysis, Multidimensional Scaling, Hierarchical Cluster Analysis, Thematic Analysis, Case Study Research, Qualitative Research, Mixed Method Research, Sustainability, Business-Society Relations, Mobility Studies, India, China, US

I confirm that I have read this submission and believe that I have an appropriate level of expertise to confirm that it is of an acceptable scientific standard.

# Response

**Published:** 2022-01-11

**Authors:** Melati Nungsari

**Affiliations:** Massachusetts Institute of Technology, USA

*Dear Reviewer,*

*We'd like to thank you for your careful read of our papers, and for your constructive comments. We're uploading a new version that addresses all of your concerns. Our responses to each of your comments can also be found below, in italics. Thank you again.*

*Authors*

 Thank you for this interesting and timely research. I hope my comments below are helpful in the final revision of your manuscript. Overall, the article is well-written, the methods sophisticated, the theoretical framework, methods, and findings clearly communicated, and the internal logic sound and easily understood. The justification for the research as outlined on pg. 3 creates an excellent tension for the importance of the research, and you make very good use of figures and tables to guide your reader throughout. Many of the sections are crisp and to the point. Thank you for your scholarly contribution!

**From a methodological perspective, I have two (small) recommendations:**

- First, the idea of data saturation in qualitative research belongs to a very specific approach in Grounded Theory. Applying it outside of the methodological frame of theoretical sampling seems attractive since it mimics the logic of representivity (i.e. we stopped conducting interviews when data saturation was reached, which implicitly suggests that all themes/characteristics have been uncovered), but of course, in the context of non-probabilistic sampling, it is entirely possible (even very likely) that sampling 34 *different* people could yield a different set of characteristics, which in turn would lead to a different 'data saturation' point and outcome. The point here is that 'data saturation' is the process through which multiple stages of theory development have been refined through multiple site visits and stages of analysis, falsification, and (re)development. It refers to an analytic process not to sampling. Applying it to denote a sampling strategy (even though done in some QL literature) is a misapplication of the concept and, given the sophistication of the methods you employ here, I would avoid making this mistake.

*Thank you for the inputs. We have removed that. *

- Second, a similar problem arises when you use the term 'correlation', which belongs to a specific set of methods and connects to a very specific language and scientific logic. In dimensional analysis, especially based on a dissimilarity matrix configuration, the relationship between themes are co-occurrences and it is more appropriate to discuss them in terms of how they are linked or associated with each other (but not correlated). I would avoid using the term correlation in an HCA framework.

*The term correlation has been replaced.*

**In terms of presenting your findings:**

- A three-step analysis can be difficult for readers to hold in their heads while following the line of argumentation. I would recommend inserting summary sections at the end of important parts to highlight to your reader what the main points were before they move on to the next section. While reading the manuscript, for example, I would have found a summary section helpful on pages 8 (before the section on 'Using MDS graphs') and 14 (before proxy agency).

*Brief summaries are added respectively.*

- The final analytic section starting on page 14 still needs some work. It is very dense and can seem list-like. While the data excerpts provide wonderful insight, it is sometimes hard to follow how the many diverse issues you introduce here come together. Overusing 'also' (it appears 17 times in this section) is an indication of the list-like nature of this part of the manuscript. Figure 4 seems to provide a good conceptual overview of these divergent issues. I wonder if you could think of a way to restructure (maybe with subheadings, a stronger thematic logic that explicitly groups different issues together, or aligning more explicitly with Figure 4) this section to make it less list-like and bring out the analytic logic of your findings. I want to stress here that my comment is not related to the content, but rather to how it is being presented.

*Thank you for the inputs. We had revised this accordingly by utilizing Figure 4 in guiding the discussion in this section.* 

- Finally, the inclusion of the news article on the UNHCR on pg. 19 undermines the systematicity of your empirical analysis. I would remove this anecdotal reference. I think your overall point will hold with the data excerpt you provide after that. 

*Noted. The reference has been removed.*

**In the discussion section:**

- It is not always clear how your recommendations are an outcome of your findings. If you strategically use summary sections earlier on in the manuscript, this would be a good time to return to them to highlight more clearly how your recommendations explicitly connect to and build from your findings presented earlier.

*Thank you for the suggestion. Changes have been made in this section.*

**In the strengths and limitation section:**

- The first sentence starts with "Using an approach that combines the consequentialist ethical framework and public health ethical principles..." seems out of place since this is the first time you are referring to this.

*It is changed to “Using Bandura’s triadic model,..”*

**General comments:**

- In both Figures 2 & 3, 'Int - Intentions', is missing from the legend.

*Added. *

- The paper could do with a final rework to remove minor spelling and grammar errors. For example, "...lack of understanding of how is human agency exercised" (pg. 1), "...not only for creating an effective and inclusive aid delivery mechanism s" (pg. 1).

*We have done this. Thank you!*

 Again, excellent research. Thank you for your contribution and good luck with your final revisions.
